# Co-occurrence of Voiding Symptoms and Flatal Incontinence in Axial Spondyloarthritis: A Cross-Sectional Study

**DOI:** 10.7759/cureus.115875

**Published:** 2026-09-06

**Authors:** Yasemin Yumusakhuylu, Hatice S Baklacioglu, Hanife Caglar Yagci

**Affiliations:** 1 Physical Medicine and Rehabilitation, Istanbul Medeniyet University, Istanbul, TUR; 2 Rheumatology, Samsun Physical Medicine and Rehabilitation Hospital, Samsun, TUR; 3 Department of Physical Medicine and Rehabilitation, Goztepe Prof. Dr. Suleyman Yalcin City Hospital, Istanbul, TUR

**Keywords:** anxiety, axial spondyloarthritis, flatal incontinence, lower urinary tract symptoms, sacropelvic dysfunction

## Abstract

Background: Axial spondyloarthritis (axSpA) is a chronic inflammatory disease of the sacroiliac joints and spine whose burden extends beyond the musculoskeletal system. Lower urinary tract symptoms are increasingly recognised in these patients, whereas anorectal symptoms have received almost no attention. Because the lower urinary tract and the anorectal continence mechanism share pelvic floor musculature and sacral neural regulation, symptoms of these two systems might be expected to co-occur, yet this has not been examined in axSpA. Anxiety and depression are also common in this population and are recognised correlates of pelvic floor symptoms in other patient groups. These considerations provided the rationale for the present study.

Objectives: This study had two objectives: first, to determine whether voiding symptoms and flatal incontinence co-occur in patients with axSpA; and second, to identify the clinical and psychological factors independently associated with flatal incontinence.

Methods: In this cross-sectional study, 137 consecutive adult patients fulfilling the Assessment of SpondyloArthritis international Society (ASAS) classification criteria for axSpA and attending two outpatient clinics between January and December 2023 were enrolled. Flatal incontinence (involuntary leakage of gas) and voiding symptoms were ascertained by structured symptom inquiry using International Continence Society (ICS) symptom definitions. Disease activity (Bath Ankylosing Spondylitis Disease Activity Index (BASDAI)), functional status (Bath Ankylosing Spondylitis Functional Index (BASFI)), psychological status (Hospital Anxiety and Depression Scale (HADS)), and inflammatory markers (erythrocyte sedimentation rate (ESR) and C-reactive protein (CRP)) were recorded. For the first objective, the association between any voiding symptom and flatal incontinence was assessed with the chi-square test, with the unadjusted odds ratio (OR) derived from the corresponding contingency table. For the second objective, patients were categorised by flatal incontinence status and multivariable binary logistic regression was performed.

Results: Flatal incontinence was reported by 54 of 137 patients (39.4%), and at least one voiding symptom by 67 of 137 patients (48.9%). Flatal incontinence was significantly more frequent among patients with voiding symptoms than among those without (41/67, 61.2% vs 13/70, 18.6%; unadjusted OR 6.91, 95% confidence interval (CI) 3.18-15.04; P < 0.001). Patients with flatal incontinence had higher BASDAI (4.84 ± 1.93 vs 3.89 ± 2.01; P = 0.007), HADS-anxiety (8.72 ± 4.06 vs 6.29 ± 3.47; P < 0.001), and HADS-depression scores (7.30 ± 3.28 vs 5.53 ± 3.88; P = 0.007), whereas ESR and CRP did not differ between groups. In multivariable analysis, only anxiety remained independently associated with flatal incontinence (adjusted OR 1.16 per HADS-anxiety point, 95% CI 1.034-1.301; P = 0.012).

Conclusions: Voiding symptoms and flatal incontinence frequently co-occurred in this axSpA cohort, and anxiety was independently associated with flatal incontinence. These hypothesis-generating findings raise the possibility of a broader sacropelvic symptom cluster in axSpA extending beyond the sacroiliac joints. Given the cross-sectional design and the absence of a control group, no causal or mediating role of psychological distress can be inferred.

## Introduction

Axial spondyloarthritis (axSpA) is a chronic inflammatory disease primarily affecting the sacroiliac joints and spine, yet its clinical burden extends substantially beyond the musculoskeletal system. Extra-musculoskeletal manifestations are well established in axSpA, and there is increasing recognition that symptom domains not captured by conventional measures of inflammation and structural damage contribute substantially to residual disease burden [1-4].

Lower urinary tract symptoms (LUTS) have emerged as a potentially underrecognised component of this burden. Increased frequencies of urinary complaints - particularly voiding and post-micturition symptoms - have been reported in patients with spondyloarthritis compared with healthy individuals and with those with other rheumatic diseases [5,6], and LUTS have been shown to correlate with functional impairment in patients with chronic lumbosacral pathology [7]. Rare cases of axSpA presenting primarily with bladder dysfunction have further highlighted that urological involvement may occasionally be the dominant clinical manifestation [8]. The mechanisms underlying the association between axSpA and LUTS remain incompletely understood. Proposed explanations include autonomic nervous system dysfunction, altered central sensory processing and central sensitization, and disturbances in sacral neural pathways involved in the coordination of detrusor and sphincter function [9,10].

The lower urinary tract and anorectal continence mechanisms constitute a closely integrated functional unit, sharing neural regulation through the pudendal nerve and sacral spinal segments (S2-S4) and depending on coordinated pelvic floor muscle activity [11]. Dysfunction affecting these shared pathways may therefore manifest simultaneously as urinary and anorectal symptoms. Despite this anatomical rationale, anorectal manifestations in axSpA remain largely unexplored, and the potential coexistence of urinary and anorectal symptoms as part of a unified sacropelvic phenotype has not been examined.

We therefore conducted this study with two objectives: first, to determine whether voiding symptoms and flatal incontinence co-occur in a well-characterised axSpA cohort; and second, to identify the clinical and psychological factors independently associated with flatal incontinence. We hypothesised that the co-occurrence of urinary and anorectal symptoms in axSpA may represent a broader pattern of sacropelvic dysfunction, potentially involving neural and functional pathways beyond structural involvement of the sacroiliac joints.

## Materials and methods

Study design and setting

This cross-sectional study was conducted between January and December 2023 at the Rheumatology Outpatient Clinic of Samsun Physical Medicine and Rehabilitation Hospital and the Physical Medicine and Rehabilitation Outpatient Clinic of Göztepe Prof. Dr. Süleyman Yalçın City Hospital, Türkiye.

Participants

Consecutive adult patients (aged 18 years or older) fulfilling the Assessment of SpondyloArthritis international Society (ASAS) classification criteria for axSpA were recruited, and 137 patients were enrolled. Exclusion criteria were a known diagnosis of inflammatory bowel disease (IBD), as concomitant IBD may directly cause bowel symptoms including flatal incontinence; a history of neurological disorders affecting bladder or bowel function; known neurogenic bladder; active urinary tract infection at the time of assessment; pregnancy; severe cognitive impairment precluding questionnaire completion; a history of pelvic or urological surgery, bladder cancer, or prostate adenoma; and other comorbid conditions unrelated to axSpA that could independently explain urinary or bowel dysfunction. No a priori sample size calculation was performed; all eligible consecutive patients presenting during the study period were enrolled. 

The sampling frame comprised all consecutive adult patients with axSpA attending these two outpatient clinics during the study period; consecutive, non-probability sampling was used, and no randomisation, stratification, or matching was applied. All patients who met the eligibility criteria during the study period were invited to participate, and those who provided written informed consent were enrolled.

Study parameters

Demographic and clinical data recorded at enrolment included age, sex, body mass index (BMI), and disease duration. Disease activity and functional status were assessed using the Bath Ankylosing Spondylitis Disease Activity Index (BASDAI) [12] and the Bath Ankylosing Spondylitis Functional Index (BASFI) [13]. Psychological status was evaluated with the Hospital Anxiety and Depression Scale (HADS) [14], comprising separate subscales for anxiety (HADS-A) and depression (HADS-D). Laboratory parameters included erythrocyte sedimentation rate (ESR) and C-reactive protein (CRP).

Urinary and bowel symptoms were ascertained through a structured symptom inquiry in which each symptom was queried directly and individually, in a manner analogous to standard clinical history taking (for example, asking whether the patient experiences leakage of gas). This inquiry was not a scored or previously validated psychometric instrument; the symptoms themselves were defined according to standardised International Continence Society (ICS) terminology [15], and the reference to ICS therefore pertains to the standardised symptom definitions rather than to a validated questionnaire. Each item was answered on a five-point frequency scale (no, rarely, sometimes, mostly, or always), a symptom was considered present if any frequency other than “no” was reported, and no summary score was calculated; each symptom was analysed as a binary variable. Flatal incontinence was defined as involuntary leakage of gas. A composite variable, “any voiding symptom,” was defined as the presence of at least one of the following: hesitancy, weak urinary stream, straining to void, post-micturition dribbling, or sensation of incomplete bladder emptying. Patients were categorised according to the presence or absence of flatal incontinence. The full list of symptom items is presented in Appendix 1.

Statistical analysis

Analyses were structured according to the two study objectives. For the first objective, the association between the presence of any voiding symptom and flatal incontinence was examined using the chi-square test, and the unadjusted odds ratio (OR) with its 95% confidence interval (CI) was calculated from the corresponding two-by-two contingency table, which is provided in Appendix 2. For the second objective, the factors associated with flatal incontinence were examined as described below.

Normally distributed continuous variables are presented as mean ± standard deviation and were compared using independent-samples t-tests. Non-normally distributed variables are presented as median (interquartile range) and were compared using the Mann-Whitney U test. Categorical variables were compared using the chi-square (χ²) test or Fisher’s exact test, as appropriate. Missing data were not imputed; analyses were conducted on available cases, and denominators are reported in the tables where data were incomplete. Binary logistic regression analysis was performed to identify independent factors associated with flatal incontinence, with age, sex, BASDAI, and HADS-A entered as covariates based on clinical relevance and univariate significance (p < 0.10). Although both HADS-A and HADS-D were associated with flatal incontinence in univariable analyses, the two subscales are strongly intercorrelated; to avoid multicollinearity, only HADS-A, which showed the stronger univariable association, was entered into the model. In view of the number of outcome events (n = 54), the number of covariates was limited to four to reduce the risk of overfitting.

In this model, adjusted ORs were obtained as exp(B), the exponentiated logistic regression coefficients. For the continuous covariates (age, BASDAI, and HADS-A), the adjusted OR expresses the multiplicative change in the odds of flatal incontinence associated with a one-unit increase in that covariate (one year of age, one BASDAI unit, and one HADS-A point, respectively), with all other covariates held constant. These estimates are model-derived and, because the covariates are continuous rather than categorical, they cannot be obtained from or displayed as a contingency table. For the single binary covariate, female sex, the adjusted OR compares women with men (reference category), and the corresponding unadjusted two-by-two contingency table is presented in Appendix 2.

Results are presented as ORs with 95% CIs. Statistical significance was defined as p < 0.05 (two-tailed). All analyses were performed using IBM SPSS Statistics for Windows, version 26.0 (IBM Corp., Armonk, NY, USA).

Ethical considerations

The study was approved by the Local Ethics Committee (Approval No: 2021/0537), and written informed consent was obtained from all participants. The study was conducted in accordance with the principles of the Declaration of Helsinki.

## Results

Co-occurrence of voiding symptoms and flatal incontinence

A total of 137 patients with axSpA were included. Flatal incontinence was reported by 54 patients (39.4%), and 67 patients (48.9%) reported at least one voiding symptom. Patients with at least one voiding symptom were significantly more likely to report flatal incontinence than those without voiding symptoms (41/67, 61.2% vs 13/70, 18.6%; OR 6.91, 95% CI 3.18-15.04; P < 0.001; Figure 1; unadjusted analysis). Constipation showed a numerically higher frequency among patients with voiding symptoms (16/67, 23.9% vs 8/70, 11.4%), although this difference did not reach statistical significance (P = 0.055).

**Figure 1:**
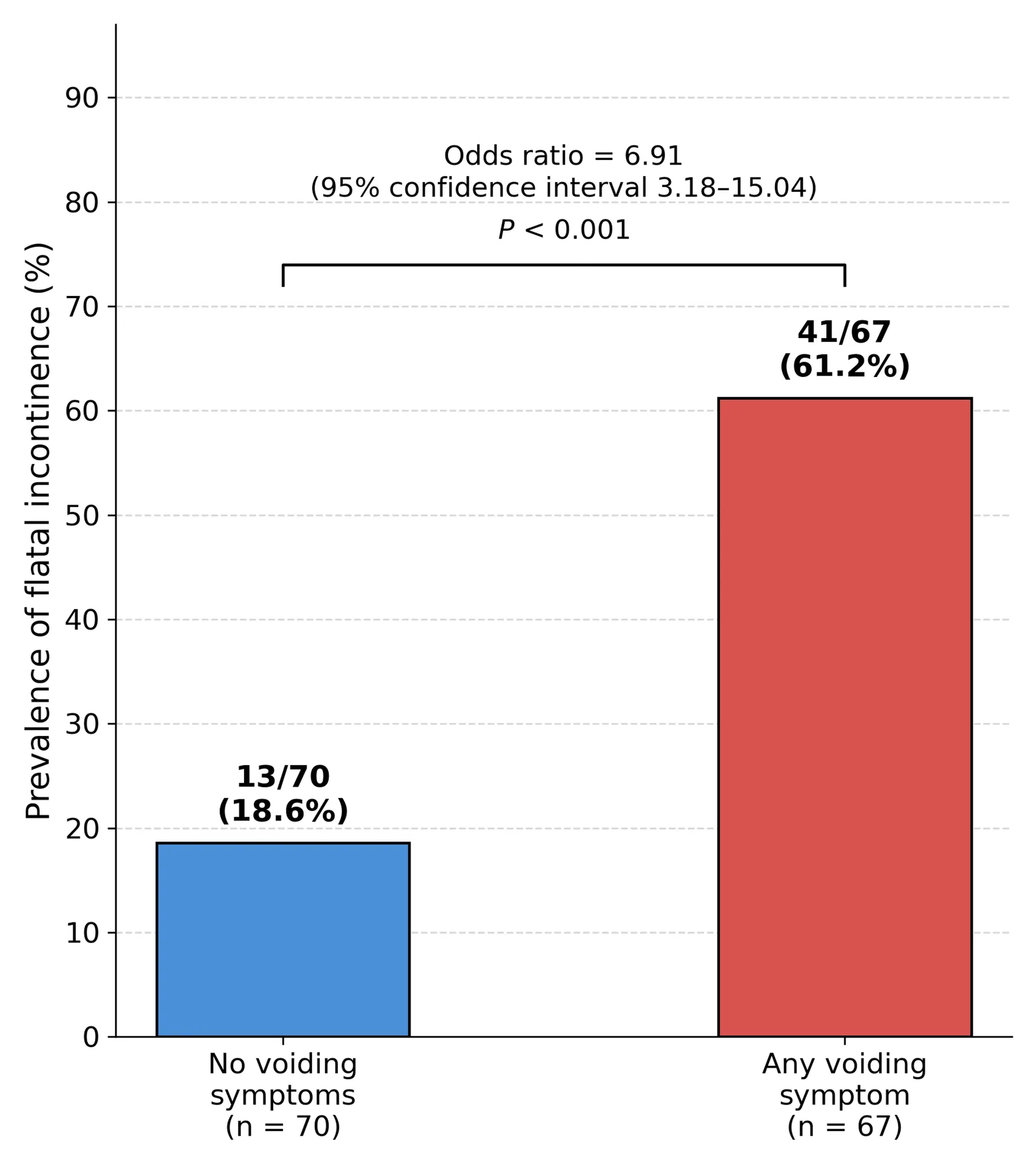
Prevalence of flatal incontinence according to voiding symptom status in patients with axial spondyloarthritis Prevalence of flatal incontinence according to voiding symptom status in patients with axial spondyloarthritis. Patients reporting at least one voiding symptom were significantly more likely to report flatal incontinence than those without voiding symptoms (41/67, 61.2% vs 13/70, 18.6%; OR 6.91, 95% CI 3.18-15.04; P < 0.001). Voiding symptoms were defined as the presence of at least one of the following: hesitancy, weak urinary stream, straining to void, post-micturition dribbling, or sensation of incomplete bladder emptying. The bars show the prevalence of flatal incontinence within each voiding symptom group, and the odds ratio displayed above the bars quantifies the strength of the association between these same two groups. Both are derived from the identical two-by-two contingency table presented in Appendix 2. The odds ratio is shown alongside the prevalences because the difference between the two prevalences alone conveys neither the magnitude of the association nor its statistical precision, whereas the odds ratio with its 95% confidence interval conveys both, and this is the effect measure reported for the first study objective in the Results. The odds ratio shown is unadjusted. OR, odds ratio; CI, confidence interval.

Characteristics of patients according to flatal incontinence status

Characteristics of patients according to flatal incontinence status are presented in Table 1. The two subgroups were comparable with respect to age, sex, BMI, disease duration, functional status (BASFI), and inflammatory markers (ESR and CRP). Patients with flatal incontinence had significantly higher disease activity (BASDAI: 4.84 ± 1.93 vs 3.89 ± 2.01, P = 0.007), anxiety (HADS-A: 8.72 ± 4.06 vs 6.29 ± 3.47, P < 0.001), and depression scores (HADS-D: 7.30 ± 3.28 vs 5.53 ± 3.88, P = 0.007). No significant differences were observed for peripheral arthritis or uveitis.

**Table 1:** Characteristics of patients with axial spondyloarthritis according to flatal incontinence status Data are presented as mean ± standard deviation or n (%). A p value < 0.05 indicates statistical significance. a Missing values present. b Calculated among patients with available data. BMI, body mass index; BASDAI, Bath Ankylosing Spondylitis Disease Activity Index [12]; BASFI, Bath Ankylosing Spondylitis Functional Index [13]; HADS, Hospital Anxiety and Depression Scale [14]; ESR, erythrocyte sedimentation rate; CRP, C-reactive protein.

Variable	Flatal incontinence (−) (n = 83)	Flatal incontinence (+) (n = 54)	p value
Demographics and body composition			
Age, years	45.1 ± 10.1	46.0 ± 10.5	0.609
Female sex, n (%)	43 (51.8)	33 (61.1)	0.284
BMI, kg/m²a	29.3 ± 7.5	29.1 ± 5.2	0.892
Disease characteristics			
Disease duration, monthsa	85.1 ± 78.0	96.0 ± 95.8	0.514
Peripheral arthritis, n (%)b	7/62 (11.3)	8/47 (17.0)	0.39
Uveitis, n (%)b	1/62 (1.6)	4/47 (8.5)	0.088
Disease activity and functional status			
BASDAI	3.89 ± 2.01	4.84 ± 1.93	0.007
BASFI	2.75 ± 1.89	2.97 ± 2.13	0.536
Psychological status			
HADS-Anxiety	6.29 ± 3.47	8.72 ± 4.06	<0.001
HADS-Depression	5.53 ± 3.88	7.30 ± 3.28	0.007
Inflammatory markers			
ESR, mm/ha	20.9 ± 15.7	18.6 ± 13.9	0.439
CRP, mg/La	8.04 ± 13.08	7.05 ± 9.62	0.671

Factors independently associated with flatal incontinence

In multivariable logistic regression analysis including age, sex, BASDAI, and HADS-A as covariates, only anxiety remained independently associated with flatal incontinence (OR 1.16, 95% CI 1.034-1.301, P = 0.012). Age, sex, and BASDAI did not reach statistical significance in the adjusted model. The overall model was statistically significant (χ² = 14.218, df = 4, P = 0.007; Nagelkerke R² = 0.133). Full regression results are presented in Table 2.

**Table 2:** Multivariable binary logistic regression analysis of factors associated with flatal incontinence in patients with axial spondyloarthritis Binary logistic regression; outcome: presence of flatal incontinence (n = 54 of 137 patients). All four covariates were entered simultaneously in a single model. Adjusted ORs are exp(B), the exponentiated regression coefficients of the model. For the continuous covariates (age, BASDAI, and HADS-anxiety), the adjusted OR represents the multiplicative change in the odds of flatal incontinence per one-unit increase in that covariate, with all other covariates held constant; for example, an adjusted OR of 1.160 for HADS-anxiety indicates that the odds of flatal incontinence increase by 16.0% for each additional HADS-anxiety point. Because these covariates are continuous, their ORs are model-derived and cannot be calculated from a contingency table. For female sex, the OR compares women with men (reference category); the corresponding unadjusted contingency table is provided in Appendix 2. A p value < 0.05 indicates statistical significance. OR, odds ratio; CI, confidence interval; BASDAI, Bath Ankylosing Spondylitis Disease Activity Index [12]; HADS, Hospital Anxiety and Depression Scale [14].

Variable	Adjusted OR (Exp(B))	95% CI	p value
Age (per one-year increase)	1.006	0.971-1.043	0.732
Female sex (vs male, reference)	0.914	0.428-1.955	0.817
BASDAI (per one-unit increase)	1.087	0.872-1.357	0.458
HADS-anxiety (per one-point increase)	1.16	1.034-1.301	0.012
Model statistics: χ² = 14.218 (df = 4), p = 0.007; Nagelkerke R² = 0.133			

## Discussion

This study found that voiding symptoms and flatal incontinence cluster in patients with axSpA, with those reporting voiding dysfunction being nearly seven times more likely to report flatal incontinence. Flatal incontinence was associated with higher disease activity and greater psychological distress in univariate analyses; however, only anxiety remained independently associated after multivariable adjustment. These findings raise the possibility that urinary and anorectal symptoms in axSpA may not be isolated manifestations but may instead reflect a broader sacropelvic dysfunction phenotype extending beyond the sacroiliac joints.

The prevalence of flatal incontinence observed in this cohort (54/137, 39.4%) is relatively high and should be interpreted in the context of the permissive symptom definition used: any reported frequency other than “no” was counted as present, and the severity and clinical impact of symptoms were not graded, nor were validated continence instruments such as the Wexner or St Mark’s incontinence scores administered. This threshold captures mild and infrequent symptoms and may therefore yield higher prevalence estimates than studies using stricter definitions or validated severity scales.

To our knowledge, this is the first study to examine the co-occurrence of voiding symptoms and flatal incontinence in axSpA. Previous work has demonstrated increased LUTS - particularly voiding and post-micturition complaints - in patients with spondyloarthritis compared with healthy controls and with other rheumatic diseases [5,6], yet anorectal symptoms have not been systematically evaluated in this population. The strong association observed here (OR 6.91, 95% CI 3.18-15.04) is consistent with the hypothesis that pelvic floor dysfunction in axSpA may involve multiple organ systems sharing common sacropelvic innervation rather than isolated bladder pathology. It should be noted that the composite “any voiding symptom” variable included several distinct lower urinary tract symptoms, which may have different underlying mechanisms. Therefore, the observed association with flatal incontinence should be interpreted cautiously and does not necessarily imply a common mechanism across all individual voiding symptoms.

There is a plausible anatomical basis for this co-occurrence. Both the lower urinary tract and anorectal continence mechanisms depend on coordinated pelvic floor activity and share neural regulation through the pudendal nerve and sacral spinal segments S2-S4 [11]. Structural involvement of the sacroiliac joints and lumbosacral spine in axSpA could theoretically influence pelvic floor-related neural pathways, although this mechanism was not directly assessed in the present study. Autonomic nervous system dysfunction, which has been documented in ankylosing spondylitis [9,10], may further contribute by impairing the coordinated reflex arcs that govern both bladder and anorectal continence. Central sensitization represents an additional plausible mechanism: recent evidence indicates that nociplastic pain processes contribute substantially to symptom burden in axSpA independently of objective inflammatory activity [16,17], and the absence of any association between flatal incontinence and ESR or CRP in the present study does not exclude the possibility that non-inflammatory mechanisms contribute to these symptoms.

The independent association between anxiety and flatal incontinence warrants particular attention. Anxiety may influence pelvic floor muscle tone, autonomic regulation, and visceral sensory processing through multiple pathways, including heightened sympathetic outflow, altered pudendal nerve afferent activity, and visceral hypersensitivity - all of which may contribute to the development or persistence of pelvic floor-related symptoms [18,19]. Conversely, bothersome anorectal symptoms may themselves amplify psychological burden, creating a self-reinforcing cycle. The cross-sectional design precludes causal inference, and both directions remain plausible. Crucially, BASDAI - which was significantly elevated in univariate analysis - was no longer independently associated with flatal incontinence after adjustment for anxiety. One speculative explanation is that disease activity influences pelvic floor symptoms partly through associated psychological distress; however, no formal mediation analysis was performed, and the cross-sectional design does not permit mediation or causal inference. This possibility would align with the growing evidence that nociplastic pain processes and central sensitization contribute substantially to symptom burden in axSpA independently of peripheral inflammatory activity [16,17].

The explanatory performance of the multivariable model deserves emphasis. The Nagelkerke R² of 0.133 indicates that anxiety and the included covariates explain only a small proportion of the variability in flatal incontinence. Determinants not captured in this study - including obstetric history, anorectal and metabolic comorbidities, medication use, and objective pelvic floor function - are likely to contribute substantially. The association between anxiety and flatal incontinence should therefore be interpreted as one component of a multifactorial symptom rather than as a dominant explanatory factor.

The sex distribution of our cohort, in which 76 of 137 patients (55.5%) were women, differs from the male predominance classically reported in radiographic axSpA. Contemporary cohorts encompassing the full axSpA spectrum, particularly non-radiographic disease, report an approximately equal sex distribution [20,21], and the referral patterns of physical medicine and rehabilitation outpatient clinics may have further increased the proportion of women in our sample. This distribution should nevertheless be borne in mind when generalising our findings to cohorts with a male predominance.

Limitations

This study has several limitations. First, and most importantly, the single-arm design without a healthy control group or a disease control group (e.g., mechanical chronic low back pain or fibromyalgia) does not allow us to determine whether the observed prevalence of flatal incontinence, or its association with anxiety, is specific to axSpA rather than a generic accompaniment of chronic painful disease; the findings should therefore be regarded as hypothesis-generating. Second, the cross-sectional design precludes conclusions regarding causality, temporal sequence, or mediation. Third, no a priori sample size calculation was performed, and the sample size was relatively small (54 outcome events), limiting the statistical power and precision of the multivariable analysis; the modest explanatory performance of the model (Nagelkerke R² = 0.133) indicates that a large proportion of the variability in flatal incontinence remains unexplained, leaving room for substantial residual and unmeasured confounding. Fourth, although patients with known inflammatory bowel disease, prior pelvic or urological surgery, and neurological disorders affecting bladder or bowel function were excluded, other conditions and exposures known to influence anorectal and bladder function - obstetric history (parity and mode of delivery), diabetes mellitus, irritable bowel syndrome, anorectal disorders, and current medications (including non-steroidal anti-inflammatory drugs, opioid analgesics, muscle relaxants, and psychotropic agents) - were not systematically recorded and could not be adjusted for. Given that more than half of the cohort were women, unmeasured obstetric factors in particular could confound the observed associations, and the association between anxiety and flatal incontinence may be partly attributable to such residual confounding. Fifth, disease activity was assessed using BASDAI only; the Ankylosing Spondylitis Disease Activity Score (ASDAS) was not collected, and BASDAI is a fully patient-reported instrument that may itself be influenced by psychological status. Sixth, the association between voiding symptoms and flatal incontinence was examined only in unadjusted analysis and may not be independent of other clinical or psychological factors. Seventh, flatal incontinence and voiding symptoms were ascertained by structured self-reported symptom inquiry using ICS-based symptom definitions rather than a formally validated instrument, and the psychometric properties of this inquiry (such as internal consistency or test-retest reliability) were not evaluated, and symptom severity and clinical impact were not graded with validated continence scales; furthermore, no objective anorectal physiological testing, anal manometry, or urodynamic studies were performed. Finally, the predominance of women in our cohort and single-country recruitment may limit generalisability to other axSpA populations.

## Conclusions

Voiding symptoms and flatal incontinence co-occurred at a high rate in this axSpA cohort, and anxiety was independently associated with flatal incontinence. These hypothesis-generating findings raise the possibility that sacropelvic symptoms in axSpA extend beyond the sacroiliac joints to involve both the urinary and anorectal continence systems, possibly through shared neural pathways; however, this mechanism, while biologically plausible, was not directly demonstrated in this study, and in the absence of a control group, disease specificity cannot be established. Routine clinical enquiry about pelvic floor symptoms and psychological well-being may nevertheless be warranted in this population. Future controlled studies incorporating objective pelvic floor assessment, comprehensive comorbidity and medication data, ASDAS-based disease activity assessment, and prospective follow-up are needed to determine whether these manifestations represent a distinct, clinically actionable phenotype in axSpA.

## Appendices

Appendix 1 

**Table 3:** Structured urinary and bowel symptom questionnaire The questionnaire was administered in Turkish; an English translation is presented. Each symptom item was answered on a five-point frequency scale (no, rarely, sometimes, mostly, or always); a symptom was considered present if any frequency other than “no” was reported. For items 6 and 10, the number of daytime and night-time voids and the number of pads used per day were also recorded. Items 14-18 constituted the composite variable “any voiding symptom,” and item 20 defined flatal incontinence.

Item	Question
1	Urine leakage when laughing, coughing, or sneezing
2	Urine leakage before reaching the toilet after a sudden urge to void
3	Urine leakage without awareness
4	Urine leakage during physical activity
5	Inability to postpone voiding
6	Number of voids: daytime (n) and night-time (n)
7	Urine leakage during sleep
8	Urine leakage during or after sexual intercourse
9	Urine leakage when nervous, hurried, or stressed
10	Wearing a pad because of urine leakage (number of pads per day)
11	Amount of urine leaked
12	Burning or pain during urination
13	Visible blood in the urine
14	Difficulty initiating urination (hesitancy)
15	Slow or weak urinary stream with dribbling
16	Straining to void
17	Dribbling of urine after voiding (post-micturition dribbling)
18	Sensation of bladder fullness after voiding (incomplete emptying)
19	Involuntary leakage of stool (faecal incontinence)
20	Involuntary leakage of gas (flatal incontinence)
21	Constipation

Appendix 2 

**Table 4:** Contingency tables underlying the unadjusted odds ratios reported in the study Panel A corresponds to the association reported in the Results and displayed in Figure 1. The unadjusted odds ratio is calculated as the cross-product ratio of this table: (41 × 57) / (26 × 13) = 6.91 (95% confidence interval 3.18-15.04; P < 0.001, chi-square test). Panel B corresponds to the only binary covariate included in the multivariable model (Table 2); its unadjusted odds ratio is (33 × 40) / (43 × 21) = 1.46, which differs from the adjusted odds ratio of 0.914 reported in Table 2 because the latter is estimated with simultaneous adjustment for age, BASDAI, and HADS-anxiety. Contingency tables are not presented for age, BASDAI, and HADS-anxiety because these covariates were analysed as continuous variables and their odds ratios are model-derived estimates per one-unit increase, which cannot be represented in a contingency table. BASDAI, Bath Ankylosing Spondylitis Disease Activity Index [12]; HADS, Hospital Anxiety and Depression Scale [14].

	Flatal incontinence present	Flatal incontinence absent	Total
Panel A. Any voiding symptom			
Any voiding symptom present	41	26	67
No voiding symptom	13	57	70
Total	54	83	137
Panel B. Sex			
Female	33	43	76
Male	21	40	61
Total	54	83	137
